# Advances in Understanding *Fusarium graminearum*: Genes Involved in the Regulation of Sexual Development, Pathogenesis, and Deoxynivalenol Biosynthesis

**DOI:** 10.3390/genes15040475

**Published:** 2024-04-09

**Authors:** Gang Niu, Qing Yang, Yihui Liao, Daiyuan Sun, Zhe Tang, Guanghui Wang, Ming Xu, Chenfang Wang, Jiangang Kang

**Affiliations:** 1 College of Plant Protection, Northwest A&F University, Xianyang 712100, China; niug@nwafu.edu.cn (G.N.); qingy@nwafu.edu.cn (Q.Y.); m18829349464@163.com (Y.L.); sdybhmx1117@163.com (D.S.); tangzhe815@163.com (Z.T.); wgh2891458@163.com (G.W.); xuming@nwafu.edu.cn (M.X.); 2 Institute of Plant Protection, Beijing Academy of Agriculture and Forestry Sciences, Beijing 100097, China; 3 College of Plant Protection, Henan Agricultural University, Zhengzhou 450002, China

**Keywords:** *Fusarium graminearum*, virulence factors, deoxynivalenol, sexual reproduction

## Abstract

The wheat head blight disease caused by *Fusarium graminearum* is a major concern for food security and the health of both humans and animals. As a pathogenic microorganism, *F. graminearum* produces virulence factors during infection to increase pathogenicity, including various macromolecular and small molecular compounds. Among these virulence factors, secreted proteins and deoxynivalenol (DON) are important weapons for the expansion and colonization of *F. graminearum*. Besides the presence of virulence factors, sexual reproduction is also crucial for the infection process of *F. graminearum* and is indispensable for the emergence and spread of wheat head blight. Over the last ten years, there have been notable breakthroughs in researching the virulence factors and sexual reproduction of *F. graminearum*. This review aims to analyze the research progress of sexual reproduction, secreted proteins, and DON of *F. graminearum*, emphasizing the regulation of sexual reproduction and DON synthesis. We also discuss the application of new gene engineering technologies in the prevention and control of wheat head blight.

## 1. Introduction

Wheat head blight, also called Fusarium head blight (FHB), is a destructive disease in wheat worldwide which leads to considerable decreases in crop productivity as well as the quality of the gathered crops due to the presence of mycotoxins in the infected grains [1,2]. The mycotoxins that form in cereals not only adversely affect the nutritional quality of the grains, but also endanger the well-being of both individuals and animals that ingest food tainted with these mycotoxins [3]. Major wheat producers situated in FHB-prone regions face a significant risk from FHB. Significant losses occur in these areas due to frequent and severe FHB outbreaks [4,5]. For decades, cereal crops in the United States have faced the most significant danger from FHB. Between 1993 and 2014, the United States experienced a staggering loss of USD 17 billion as a result of FHB impacting wheat [3,4,6]. Since 1950, China has experienced 30 FHB epidemics, following more than 10% loss of the total acreage every time. The major epidemic in 2012 led to the destruction of around 10 million hectares of wheat cultivation and a loss of over 2 million tons in yield [2]. Since 2016, FHB has been gradually spreading northwards and is increasingly becoming a common disease affecting wheat in the Huang Huai Plain (HHP) of China [7]. The occurrence of FHB epidemics has been influenced by changes in the planting conditions and the rise in global temperatures [8]. Fluctuations in temperature and humidity significantly influence the spread of FHB infection [2]. 

The infection process of *F. graminearum* in wheat starts when ascospores are released from the perithecia and then land on wheat spikelets through the air [9]. Except for the ascospore, there is another spore type, conidia, and both of them play key roles in disease initiation and propagation. However, it is believed that ascospores, which are forcefully released into the atmosphere, act as the primary inoculum of infection in the disease cycle [10]. Hence, the process of sexual maturation and the release of ascospores play a crucial role in the survival of fungi and the onset of diseases [10]. The ascospores adhere to the surface of the host and initiate the growth of germ tubes. Subsequently, these germ tubes transform into distinct non-branching filaments known as runner hyphae (RH). Multicellular infection cushions (IC) differentiate from RH; they penetrate the plant cuticles and generate multiple sites for infection initiation [11,12,13]. Following the initial infection, the fungus spreads into the inner tissues of the growing grains using the invasive hyphae (IH), which extend throughout the spikelet, reaching the rachial node. Eventually, FHB symptoms become evident in various spikelets as the IH spreads upwards or downwards along the rachis [3,14,15,16]. Through RNA-seq and transcriptome analysis, it was discovered that infection-related genes were up-regulated in the IC in comparison to the RH. These genes encompassed carbohydrate-active enzymes (CAZymes), potential effectors, and clusters of genes associated with secondary metabolism [17]. The existing evidence proves that deoxynivalenol (DON), biosynthesized by *F. graminearum*, is crucial for the spread of fungus from spikelet to rachis during infection [18]. 

It is not possible to effectively manage FHB by relying on a single control strategy due to their individual limitations. In practical terms, employing a combination of control strategies, including cultural practices, biological methods, chemical treatments, and host plant resistance, can contribute to effectively managing FHB to some extent [3]. Moreover, cultivars with strong resistance would offer the most effective approach to decrease FHB outbreaks [19]. Identifying genes associated with FHB resistance and incorporating them into the breeding of disease-resistant varieties is an effective and cost-efficient solution for managing FHB [19]. Presently, there is a considerable amount of documented quantitative trait loci (QTL) or genes that provide resistance against FHB, and relevant reviews are available on the resistance genes and control strategies for FHB [3,19]. In this review, we focus on two aspects closely related to *F. graminearum* infection and FHB occurrence: the primary inoculum and the virulence factors (secreted proteins and deoxynivalenol) during the infection process. Additionally, we discuss new technologies related to FHB prevention and control.

## 2. Sexual Reproduction of *F. graminearum* Provides the Primary Inoculum for FHB

Like other eukaryotic creatures, fungi rely on sexual reproduction to promote genetic variation and eliminate detrimental mutations [20]. Sexual development is essential for the disease cycle of FHB. In diseased wheat, the initial stage of perithecium, along with the binucleate hyphae from which they originate, are linked to the plant’s stomata and silica cells; these structures serve as overwintering sites. In the field, the perithecia are short-lived; *F. graminearum* depends on forcibly ejecting ascospores from sexual reproduction to infect wheat flowers. The primary inoculum of infection for the disease is the airborne ascospores [21]. Therefore, sexual reproduction of *F. graminearum* provides the primary inoculum for FHB.

### 2.1. The Sexual Development Processes of F. graminearum

**Figure 1:**
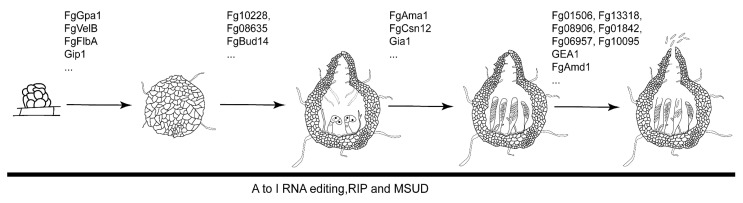
**Genes involved in the regulation of sexual reproduction in *F. graminearum*.** Proteins such as G protein-coupled receptors Gip1, Gα subunits of heterotrimeric G proteins Gpa1, RGS (regulator of G protein signaling) proteins FgFlbA, and components of velvet protein complex FgVelB are essential for the formation of perithecium. FgBud14, Fg10228, and Fg08635 play critical role in the ascus development. The proteins FgAma1, FgCsn12, FgGia1, and others have varying effects on the development of the ascus and ascospore. FgAmd1; Gea1; and protein kinases Fg01506, Fg13318, Fg08906, Fg01842, Fg06957, and Fg10095 have been shown to be essential in ascospore release.

The sexual development of *F. graminearum* starts with the formation of hyphae that contain binucleate cells, which have two genetically identical nuclei and are responsible for sexual reproduction. The binucleate cells then develop into small, coiled cells known as fruiting body initials. In culture, the initial fruiting bodies progress without interruption and ultimately transform into structures resembling flasks, which are referred to as perithecia [9,22]. Perithecia have different tissue types that are produced at specific stages of perithecium development, including the formation of perithecium initials, the outer wall, paraphyses, asci, and ascospores [21]. The asci extend vertically within the perithecium, producing ascospores in two rows, each containing eight ascospores per ascus. The ascospores are discharged via an opening situated at the tip of the ascus, which traverses the ostiole [21]. Sexual reproduction in ascomycetes is regulated by transcription factor genes (TFs) at the mating type (MAT) locus. In *Saccharomyces cerevisiae*, MAT-encoded TFs regulate genes involved in pheromone production and receptor activity [20]. When pheromones bind to the Ste2 and Ste3 G protein-coupled receptors (GPCRs), they trigger the pheromone response pathway by activating the downstream cascade involving Ste11, Ste7, and Fus3/Kss1 [20]. MAT transcription factors in *F. graminearum* are not necessary for the early stages of mating, but they are essential for the formation and expansion of dikaryotic hyphae as well as the later phases of sexual reproduction [23]. Nonetheless, the presence of pheromones and pheromone receptors does not play a crucial role in the sexual reproduction of *F. graminearum* [24]. The factors that trigger the formation of croziers, meiosis, and ascus development in filamentous ascomycetes are still unknown [25]. However, previous research has discovered different genes that impact sexual processes in *F. graminearum*, including non-pheromone GPCRs [26] (Figure 1).

### 2.2. Genes Involves in the Formation of Perithecia

The development of perithecia is closely connected to intracellular signaling. Heterotrimeric G proteins are highly conserved in model filamentous fungi. The essential components of the G protein signaling complex include G protein-coupled receptors, G proteins (comprising Gα, Gβ, and Gγ subunits), and downstream effectors [27]. A non-pheromone GPCR Gip1 (Fg05239) has been identified as crucial for perithecium formation in *F. graminearum*. Δ*fg05239* mutants are capable of forming protoperithecia but cannot progress to develop mature, melanized perithecia [26]. A recent study verified that Gip1 orthologs have a conserved role in the development of perithecium in both heterothallic and homothallic species [25]. Deletion of the Gα subunits Gpa1 in heterotrimeric G proteins leads to defects in the development of the perithecium in *F. graminearum*, indicating the essential role of *GPA1* in regulating sexual reproduction [28]. Regulators of G protein signaling (RGS) are crucial in the regulation of heterotrimeric G protein signaling [29]. FgFlbA is an RGS protein that interacts with the Gα subunit. The *fgflbA* mutants are unable to produce perithecia through self-fertilization, resulting in the loss of their ability for female fertility [30]. 

The formation of perithecium is influenced not only by the G protein signaling complex, but also by various downstream signaling pathways. Eukaryotic organisms heavily depend on mitogen-activated protein kinase (MAPK) pathways to respond to both abiotic and biotic stresses [31]. *F. graminearum* possesses three MAPKs (Gpmk1/Map1, Mgv1, and FgHog1) [13]. *MGV1* acts as the MAPK pathway for the cell wall integrity (CWI). Mutants lacking *MGV1* are unable to produce perithecia under selfing conditions. A recent study examined the composition and role of the striatin-interacting phosphatases and kinases (STRIPAK) complex in *F. graminearum*. It was found that STRIPAK mutants did not show any perithecia formation in the same environment as the wild type. Additional discoveries indicated that the STRIPAK complex manages the coordination of cell wall integrity signaling to control the fungal growth and virulence of *F. graminearum* [32]. Deletion of another MAPK *GPMK1* also resulted in defects in sexual reproduction, Δ*gpmk1* mutants failed to produce any perithecia [33]. FgSte12 and FgMcm1 are two transcription factors downstream of Gpmk1. The deletion mutant of *FgSTE12* produced significantly less perithecia than the wild type [34]. Loss of *FgMCM1* led to infertility, as well as a notable decrease in virulence and DON production [35]. The involvement of RAS2, a GTPase, in the activation of Gpmk1 has been confirmed to be crucial for sexual reproduction in *F. graminearum*, as evidenced by the mutant defect of *ras2* in female fertility [36]. FgHOG1 is crucial for infection in *F. graminearum*, while its ortholog in *Magnaporthe oryzae* is not required for virulence. The FgSsk2–FgPbs2–FgHog1 MAPK cascade was also found to be essential for female fertility [37]. To determine the MAPK-less effects in *F. graminearum*, deleted mutants of all three MAPK genes were generated in a study. The *gpmk1 mgv1 fghog1* triple mutants were unable to engage in sexual reproduction as a result of the loss of female fertility [38]. A systematic study of protein kinases in *F. graminearum* showed that 20 mutants were unable to produce perithecia. Among these, six mutants belonged to the Mgv1 and Gpmk1 MAPK pathway [39]. These results indicate that the three MAPK pathways are indispensable for the development of perithecia in the sexual reproduction process of *F. graminearum*. Inhibition of another important signaling pathway in *F. graminearum*, the cAMP-PKA signaling pathway, also affects the formation of perithecia, as evidenced by the blocked perithecium development observed in the *pkr* (the regulatory subunit of PKA) mutant [40].

In addition to these signaling pathways, numerous other genes exert pivotal functions in the development of perithecia in *F. graminearum* (Figure 1). The *FGK3* gene, which encodes glycogen synthase kinase, was found to be a crucial determinant of virulence in *F. graminearum* [39]. The Δ*fgk3* mutant resulted in the inability to generate perithecia and protoperithecia, indicating the vital involvement of FGK3 in the initial stages of sexual development in *F. graminearum* [41]. The velvet protein complex formed a heterotrimeric complex comprising VelB–VeA–LaeA proteins. In *F. graminearum*, the *fgvelB* mutant failed to produce fruiting bodies [42]. FgEps1 is a protein disulfide isomerase of *F. graminearum*, and it was found that Δ*fgeps1* produced no perithecia on the medium [43]. The AP1 complex, a clathrin adaptor that is highly conserved, includes FgAP1σ as one of its subunits in *F. graminearum*. The absence of FgAP1σ in *F. graminearum* resulted in the complete elimination of perithecia formation [44]. The RNA lariat debranching enzyme Dbr1 is essential for intron turnover. *fgdbr1* mutants produced limited immature perithecia in *F. graminearum* [45]. *Fgporin* was characterized as a yeast mitochondrial porin orthologue in *F. graminearum*, and the Δ*fgporin* mutant was unable to generate perithecia until 20 days after fertilization [46]. FgErv14 was identified as an endoplasmic reticulum (ER) cargo receptor in *F. graminearum*. Two weeks after fertilization, the Δfgerv14 mutant showed a complete absence of perithecia production [47]. Systematic investigation of Phox homology domain-containing proteins in *F. graminearum* revealed that FgBem1 plays a crucial role in both sexual development and virulence. The *fgbem1* mutant was unable to form perithecium [48]. Sgh1 is a serine/arginine (SR)-like protein that participates in pre-mRNA processing in *F. graminearum.* The Δ*sgh1* mutant did not produce any protoperithecia or perithecia on mating plates [49] FgExosc1 and FgExoscA are part of the RNA exosome complex in *F. graminearum*. The deletion mutant of *Fgexosc1* was unable to form perithecia. Although the deletion mutant of *FgexoscA* exhibited normal perithecia formation, it showed a significant reduction in the quantity of ascospores generated compared to the wild-type strain PH-1 [50] (Supplementary Table S1).

The deletion defects of some genes are manifested by reduced production or delayed maturation of perithecia rather than no perithecia. *FgSFL1* and *FgATF1* were identified as downstream effectors of the PKA signaling pathway and the HOG1 signaling pathway, respectively. Deletion mutants of *fgsfl1* exhibited a decrease in the quantity of perithecia formed, while mutants of *fgatf1* exhibited delayed perithecium development [51,52]. *MES1* is a gene involved in cell-surface organization, and *mes1* mutants consistently produced fewer perithecia in *F. graminearum*. Although the *mes1* mutants showed a decrease in perithecium formation, the ascospores they produced were morphologically indistinguishable from those produced by the wild-type strain PH-1 [53]. FgMet3 and FgMet14 are two proteins related to the synthesis of cysteine and methionine in *F. graminearum*. The progression of perithecium formation was delayed in the *fgmet3* and *fgmet14* mutants compared to PH-1 [54]. FgCapA and FgCapB, the two actin-capping proteins (CAPs), were identified as two components of toxisomes. The *ΔFgcapA* and *ΔFgcapB* mutants exhibited a reduced number of perithecia in comparison to the wild type [55]. The deletion mutants of these genes all affect perithecia formation, but they may have roles beyond the perithecia formation stage. To confirm this, stage-specific silencing experiments are necessary.

### 2.3. Genes Involves in Ascosporogenesis

The perithecia contain numerous asci, which are elongated sac-like structures that contain eight haploid ascospores each. The asci are formed through meiosis [9]. Although the regulatory mechanism of ascosporogenesis in filamentous fungi remains unclear, existing research suggests that the regulation of meiosis and ascosporogenesis is closely associated with surface receptors and downstream signaling pathways. Recent research has shown that Gia1, a G protein-coupled receptor that is not involved in pheromone signaling, regulates the initiation of meiosis and ascosporogenesis through the Gpmk1 MAPK signaling pathway in *F. graminearum* and other filamentous ascomycetes [25]. FgSwi6 and Fgp1 are two transcription factors downstream of the CWI signaling pathway in *F. graminearum*. Δ*fgswi6* show reduced perithecium production and size, as well as a decreased production of asci and ascospores [56]. The perithecia of Δ*fgp1* resembles the wild type, but ascospore formation is delayed by one week, and only a limited number of ascospores are released from the perithecia [57]. *CPK1* and *CPK2*, as regulatory subunits of PKA protein, function in the cAMP-PKA signaling pathway. The *cpk1* mutant shows deficiencies in ascospore maturation and release while the *cpk2* mutant does not show any noticeable phenotypes [58].

The ascosporogenesis is also influenced by genes other than the signaling pathway (Figure 1), such as the previously mentioned *FgExoscA* [50]. The systematic analysis of kinases found that deletions of protein kinases *FgDBF1* and *FgSWE1* were shown to be aborted in ascus development, while mutants of Fg08468, Fg07344, Fg06878 (Cmk1/2), and Fg10095 showed significant decreases in ascospore formation [39]. Deletion of *FgKIN1*, a gene encoding MARKs (microtubule affinity-regulating protein kinases), led to decreased virulence and compromised ascospore germination and dissemination [59]. The COP9 signalosome (Csn) complex is a highly conserved protein complex that plays a role in regulating various essential cellular processes across evolution [60]. The subunit of COP9 signalosome FgCsn12 is also involved in regulating ascosporeogenesis and sexual development [61]. *FgBUD14* encodes a protein with homology to yeast Bud14, and deleting *FgBUD14* greatly decreases the formation of croziers and the development of asci [62]. *FgLEU1* encodes an isopropylmalate isomerase in *F. graminearum*. The Δ*leu1* mutant fails to generate ascospores [63]. 

In the life cycle of *F. graminearum*, the discharge of ascospores is crucial for the survival of the fungus and the initiation of disease [64]. The release of ascospores is driven by the turgor pressure created through ion fluxes, particularly potassium (K+) and calcium (Ca2+), along with the buildup of mannitol [64]. High humidity levels and low air temperatures have been proven to be linked to ascospore discharge [65]. Apart from physiological factors, certain genes are also closely related to the discharge of ascospores (Supplementary Table S1). The systematic analysis of kinases found that deletions of protein kinases Fg01506, Fg13318, Fg08906, Fg01842, Fg06957, and Fg10095 were shown to be defective in ascospore release [39]. *ROA* (ORF round ascospore) has been identified as a new gene that performs various functions in preserving the correct morphology and release of ascospores in *F. graminearum* [66]. The deletion of the calcium ion channel gene *CCH1* was found to stop ascospore discharge while not influencing spore or ascus morphology [67]. FgSRP1, a serine/arginine-rich protein, is crucial for conidiation, pathogenesis, alternative splicing, perithecium pigmentation, and ascospore discharge [68]. lncRsp1 is a long noncoding RNAs positioned +99 bp upstream of the putative sugar transporter gene, *FgSP1*. Both Δ*lncRsp1* and Δ*Fgsp1* mutants exhibit normal growth and conidiation, but show deficiencies in ascospore discharge and pathogenicity on wheat coleoptiles [69]. *FgATF1* is a stress-related transcription factor gene. A mutant of *fgatf1* exhibits a notable decrease in virulence and a delay in ascospore release [70]. *GEA1* is a gene that plays a critical role in the development of the ascus wall in *F. graminearum*. Deleting *GEA1* leads to the formation of abnormal ascus walls that collapse before ascospore discharge [71].

### 2.4. Epigenetic Regulation during Sexual Reproduction in F. graminearum

Sexual reproduction involves a complex interplay of genetic and metabolic processes, which are likely to be finely regulated in terms of timing and location at every stage of sexual development [72]. In this process, epigenetic regulation also plays an important role, such as repeat-induced point mutation (RIP), meiotic silencing by unpaired DNA (MSUD), and A-to-I RNA editing [73,74,75,76]. RIP is a genome mutation process specific to certain fungal taxa, targeting repeated DNA sequences. Before meiotic prophase, it identifies and alters duplicated transposable elements, resulting in the formation of transposons that are not functional [77]. The mechanism of RIP remains unknown, but one common result of its happening is the occurrence of methylation. DNA sequence analysis indicates that the methylated portion of the genome primarily comprises remnants of transposons that underwent RIP [78]. The genome of *F. graminearum* is characterized by a scarcity of repetitive DNA sequences and a notable absence of active transposable elements when compared to other similar fungi, largely due to its homothallic nature and the presence of the RIP system during each meiosis [76]. Following karyogamy, unpaired DNA during meiosis leads to the silencing of all DNA sequences homologous to it, including genes that are already paired; this mechanism is referred to as MSUD [79]. MSUD functions by recognizing and inhibiting the replication of repetitive sequences, thus averting the activation of transposons in meiotic cellular division [79]. Although *F. graminearum* is homothallic, MSUD is still active in this species, albeit at a lower level compared to *Neurospora crassa*. The reduced activity of meiotic silencing in *F. graminearum* seems to be an evolutionary adaptation to minimize fitness costs during sexual reproduction [80]. A-to-I RNA editing is a crucial post-transcriptional alteration that transforms adenosine (A) into inosine (I) in RNA molecules [81]. The initial discovery of fungal A-to-I mRNA editing occurred in the mRNA of Puk1 within *F. graminearum* [74]. *PUK1* has a distinct function in the formation and discharge of ascospores [74]. In addition to *PUK1*, several genes related to A-to-I editing have been discovered in *F. graminearum. FgAMA1* is a gene that encodes a meiosis-specific activator of APC/C31, which is a protein complex that regulates cell cycle progression and chromosome segregation during meiosis. It has been demonstrated that the A-to-I RNA editing of *FgAMA1* is important for ascospore formation and discharge in *F. graminearum* [82]. *AMD1* is a gene with a premature stop codon that relies on A-to-I RNA editing to produce a complete functional protein. AMD1 might have a crucial function in preserving ascus wall integrity during ascus maturation [83]. During sexual reproduction of *F. graminearum*, *FgBUD14* plays crucial roles in ascus development, with its transcripts undergoing both specific alternative splicing and RNA editing [62]. Feng et al. conducted a pioneering study that revealed key RNA sequence and structure features influencing editing. Their research identified cis-sequence elements with different roles in editing specificity and efficiency in *F. graminearum* [84]. The study conducted by Xin et al. on missense editing sites provided compelling experimental proof of the adaptive benefits of RNA editing in fungi and possibly in animals [85]. A recent study indicated that restorative RNA editing functions as an adaptive mechanism that allows for the reconciliation of genetic trade-offs [86].

In addition to these three mechanisms, the sex-induced RNA interference (RNAi) mechanism has also been identified as playing crucial roles in sexual reproduction of *F. graminearum* [87]. RNA interference (RNAi) is a preserved process activated by double-stranded (ds)RNA. It offers defense against external genetic material, controls gene activity that codes for proteins during and after gene expression, and maintains genome stability by suppressing transposons [88,89,90]. In this process, Dicers, which belong to the RNase III family of nucleases, cleave double-stranded RNA (dsRNA) precursors to produce siRNA and miRNA duplexes [91]. The resulting siRNA or miRNA duplexes are then integrated into an RNA-induced silencing complex (RISC), where Argonaute serves as the central component and acts as an sRNA-guided endonuclease [91]. RISC is activated following the removal of the passenger strands of sRNA duplexes. The guide RNA integrated into RISC is subsequently employed to identify matching mRNA for suppression via mRNA degradation or inhibition of translation [92,93]. *F. graminearum* has two Dicers and two Argonautes. Research has revealed that the regulation of Argonaute genes is influenced by the mating-type gene and is crucial for sexual maturation in *F. graminearum* [94]. Son et al. confirmed that *F. graminearum* employs the ex-siRNA-mediated RNAi pathway exclusively for sexual development, which is mainly regulated by *FgDCL1* and *FgAGO2* [87]. Meanwhile, through the use of sRNA and transcriptome sequencing, 143 new microRNA-like RNAs (milRNAs) were identified in wild-type perithecia, with the majority of them being dependent on *FgDCL1*. These milRNAs specific to perithecia could potentially be involved in sexual development, as they are predicted to target 117 genes [95].

## 3. Virulence Factors Secreted by *F. graminearum* during Wheat Infection

### 3.1. F. graminearum Secretes a Variety of Enzymes and Effectors to Facilitate Infection

**Figure 2:**
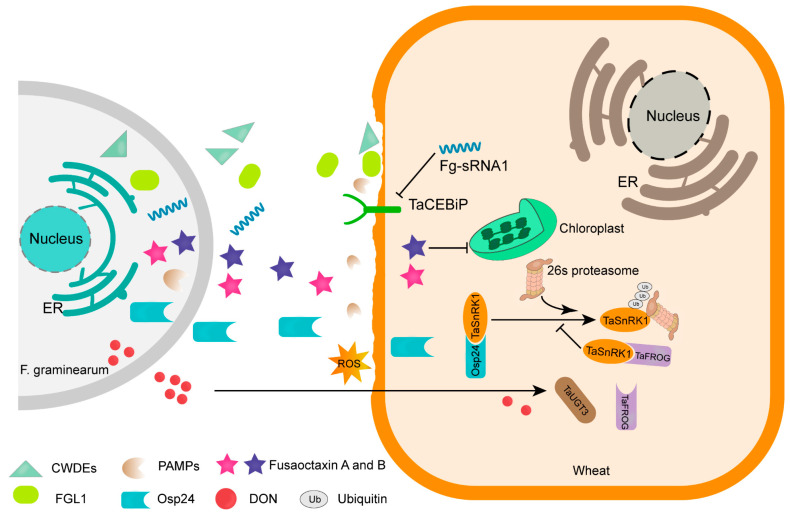
**Model of interaction between virulence factors secreted by *F. graminearum* and targets**. Fg-sRNA1 interacts with chitin elicitor binding protein (TaCEBiP). Cell-wall-degrading enzymes (CWDEs) secreted by *F. graminearum* degrade plant tissues. Fusaoctaxin A and B alter chloroplast localization and distribution to facilitate infection. Lipase FGL1 suppresses callose deposition. The cytoplasmic effector Osp24 competes with the resistance protein TaFROG for binding with the immunity-related kinase TaSnRK1a, and thereby accelerates TaSnRK1a degradation. TaFROG and the UDP-glycosyltransferase TaUGT3 contribute to host resistance to DON.

Pathogenic fungi employ a diversity of small secreted proteins (SSPs) or molecules that modulate host cell structure, metabolism, defense responses, and other cellular processes to facilitate infection (Figure 2) [96]. An analysis comparing the transcriptome of wheat tissues infected by *F. graminearum*, with and without symptoms, demonstrated a significant up-regulation of genes encoding cell-wall-degrading enzymes (CWDEs) in both asymptomatic and symptomatic wheat tissues. This suggests the vital importance of these genes in various stages of infection [97]. In the dicot *Nicotiana benthamiana*, two glycoside hydrolase 12 (GH12) family proteins, Fg05851 and Fg11037, are recognized as targets of LRR receptor-like protein response to XEG1 (RXEG1). Introducing RXEG1 into wheat enhances resistance to *F. graminearum* by targeting Fg05851 and Fg11037, leading to reduced mycotoxin levels in wheat grains [98]. Enzymes such as tomatinase-like enzyme, arabinanase, catalase-peroxidase, and ribonuclease, encoded by *FgTOM1*, *ARB93*B, *KATG2,* and *Fg12,* respectively, were identified as pathogenicity determinants contributing to *F. graminearum* virulence [99,100,101,102]. In addition to CWDEs, other enzymes such as lipases and proteases are also secreted into the extracellular space to breach the primary plant cell defense barrier [103]. FGL1, a lipase secreted by *F. graminearum*, acts as a virulence factor facilitating pathogen infection through its enzymatic activity. The *fgl1* mutant elicits a strong wheat defense response involving callose deposition [104]. 

Additionally, various works have found that *F. graminearum* deploys many effectors for suppressing host immunity and promoting infection in the process of the interaction between the pathogen and wheat [105,106,107]. The orphan secreted protein Osp24 suppresses Bax- or INF1-induced cell death, and the osp24 deletion mutant affects the expansion of invasive hyphae in wheat rachis tissues. Osp24 interacts with TaSnRK1α and promotes its degradation by facilitating TaSnRK1α binding with ubiquitin-26S proteasomes, thereby reducing wheat’s resistance to Fusarium head blight [108]. A small secreted protein gene was found to have increased expression during infection of wheat heads by *F. graminearum*. Deleting Fg02685 slowed down expansion of *F. graminearum* in wheat spikes. The 32-amino-acid N-terminus peptide of Fg02685 has been shown to play a key role in inducing oxidative burst, callose deposition, and activating MAPK signaling in plants [109]. *F. graminearum* secretes a group of cysteine-rich proteins common in the fungal extracellular membrane (CFEM) domain that specifically target the interacting protein of ZmWAK17, a receptor kinase associated with the cell wall. This interaction has a negative regulatory effect on ZmWAK17-mediated immunity [110]. 

### 3.2. DON Is a Crucial Virulence Factor Necessary for the Proliferation of Infections on Wheat Heads

The release of mycotoxins by FHB pathogens is a significant concern, as it can have detrimental effects on wheat grains. These mycotoxins not only impact the nutritional quality of the grains, but also pose a risk to the health of humans and livestock who consume food contaminated with mycotoxins [2]. As the most common mycotoxin in cereal grains worldwide, DON inhibits protein synthesis and causes various harmful effects in mammals, such as emetic effects, anorexia, and immune dysregulation [111,112]. DON is also a critical virulence factor of *F. graminearum* [113]. DON biosynthesis is strongly induced when *F. graminearum* infects spikelets of wheat and spreads throughout the entire head [114]. Deleting the initial trichodiene synthase gene, *TRI5,* leads to decreased virulence. Δ*tri5* mutants are restricted to the inoculated wheat spikelets and unable to pass through the rachis node [115]. 

### 3.3. Genes Involves in DON Production

**Figure 3:**
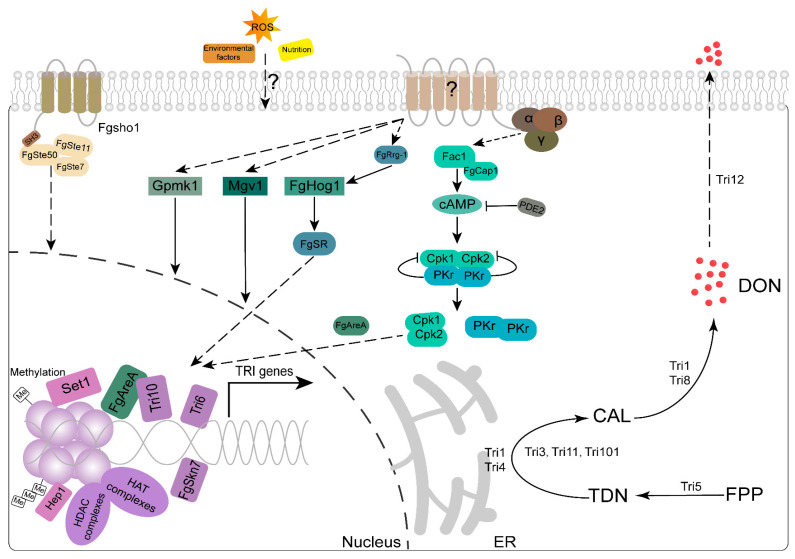
**Genes and environmental factors involved in the regulation of DON synthesis**. Factors of the environment, such as oxidative stress and nutrition, induce DON synthesis during *F. graminearum* infection. The transmembrane protein FgSho1 is required for deoxynivalenol (DON) biosynthesis in *F. graminearum*. FgSho1 physically interacts with the MAPK module FgSte50-Ste11-Ste7. Gpa1 and Gpb1 act as negative regulators of DON production. FgCap1 interacts with adenylate cyclase Fac1 and modulates DON production via cAMP signaling. Cpk1 is the major PKA catalytic subunit gene involved in DON synthesis. The cAMP phosphodiesterase Pde2 and the regulatory subunit of PKA (PKR) also negatively regulate DON production. DON biosynthesis is blocked when all three MAPKs are deleted in *F. graminearum*. Tri6 activates the expression of most genes in the DON biosynthetic pathway. *TRI10* has been suggested to act upstream of *TRI6*. AreA mediates the regulation of deoxynivalenol (DON) synthesis by cAMP signaling. AreA is involved in the transcriptional regulation of *TRI* genes through its interaction with Tri10. FgSR and FgRrg-1 are closely related to the synthesis of DON and the expression of DON synthesis-related genes. Deleting the heterochromatin protein Hep1 suppresses the expression of *TRI5* and *TRI6*. FgSet1-mediated histone 3 lysine 4 methylations (H3K4me) modulate the expression of *TRI* genes. Histone acetyltransferase (HAT) and histone deacetylase (HDAC) complexes have been shown to be associated with DON synthesis. Tri5 cyclizes farnesyl pyrophosphate (FPP) to trichodiene (TDN). TDN is then converted to calonectrin (CAL) by nine reactions sequentially catalyzed by Tri4, Tri101, Tri11, and Tri3. CAL is hydroxylated by Tri1 and deacetylated by Tri8, leading to the formation of either 3-ADON or 15-ADON, followed by DON. Tri1 and Tri4 are localized to toxisome which is formed through remodeling of the endoplasmic reticulum (ER) and involved in the early and late steps of DON biosynthesis. Tri12 facilitates the transport of trichothecene metabolites across a membrane barrier and confers toxin resistance.

The 15 *TRI* genes encode the necessary biosynthetic enzymes for the production of trichothecene. Following the discovery of the *TRI5*, which codes for trichodiene synthase, a total of 10 biosynthesis genes were found within the *TRI5* gene cluster. *TRI101, TRI1,* and *TRI16* were discovered situated outside the gene cluster of *TRI5* [116,117]. *TRI6* and *TRI10* function as global transcriptional regulators within the *TRI* gene cluster, stimulating the transcription of additional *TRI* genes [118,119]. A recent study discovered that *TRI10* and *TRI6* mutually control each other’s expression and play a crucial role in inhibiting the expression of a long non-coding RNA (RNA5P) [120]. In addition to *TRI* genes, the regulation of DON production is also related to intracellular signaling (Figure 3). The target of rapamycin (TOR) pathway is a conserved signaling mechanism found in organisms ranging from yeast to humans. It serves as a connection between external stimuli, such as nutrients and growth factors, and internal processes involved in development and metabolism [121]. TOR may also regulate DON production via biogenesis of lipid droplets in *F. graminearum* [117,122]. Deletion of *CPK1* results in a significant decrease in DON synthesis, while the *cpk2* mutant shows no observable phenotypes [58]. Deletion of *PDE2* encoding cAMP phosphodiesterase and *PKR* leads to an elevation in DON production [40,119]. The adenylate-binding protein FgCap1 interacts with adenylate cyclase Fac1, influencing DON production through cAMP signaling, and is under feedback regulation by *TRI6* [123]. In *F. graminearum*, the CWI signaling pathway comprises FgBck1, FgMkk1, and FgMgv1 as the MAPK components. The Δ*fgmgv1* mutant exhibits a substantial decrease in trichothecene accumulation in wheat heads after inoculation, as well as reduced levels of ΔFgBck1 and ΔFgMkk1 [39,124,125]. Deletion mutants of the FgSte11-Ste7-Gpmk1 signaling cascade lead to decreases in the expression of *TRI* genes and reduced DON production [39,126,127]. Deletion of the response regulators FgOs1 and FgRrg1, as well as the response factor FgAtf1 in the HOG pathway, results in a significant decrease in DON production [37,52,128,129,130].

In recent years, many other genes controlling DON synthesis beyond *TRI* genes and signaling pathways have been identified (Supplementary Table S2). Under the induction conditions of DON, transcription factor FgStuA recruits the Spt-Ada-Gcn5-Acetyltransferase (SAGA) complex to the *TRI6* promoter, leading to increased *TRI6* transcription [131]. FgPex13 and FgPex14 are peroxisomal docking machinery components. Δ*fgpex13* and Δ*fgpex14* cause a deficiency in acetyl-CoA, which is critical for trichothecene biosynthesis; as a result, the production of deoxynivalenol (DON) decreases [132]. The subtilisin-like protease FgPrb1 and long non-coding RNA (lncRNA) lncRsp1 both exert an influence on DON synthesis [69,133]. Moreover, epigenetic mechanisms also play a crucial role in regulating DON production. These mechanisms involve the regulation of heterochromatin, histone methylation, and acetylation [13,117]. Various proteins such as Hep1, Kmt6, FgGcn5, Elp3, and FgSas3, which are associated with heterochromatin, histone methylation, and acetylation, have been identified to be involved in regulating the expression of *TRI* genes and the biosynthesis of deoxynivalenol [13,134,135]. The inhibitor of growth (ING) proteins Fng1 and Fng3, which are associated with histone acetyltransferase (HAT) and histone deacetylase (HDAC) complexes, are required for the biosynthesis of DON [13,136,137]. 

*TRI* genes are highly expressed and translated into proteins under DON induction conditions. A portion of these proteins are situated in a perinuclear organized smooth endoplasmic reticulum (OSER), the site where DON biosynthesis takes place, commonly known as the ‘toxisome’ [138]. In recent years, some genes related to the formation of toxisomes have been discovered. *FgSUR2* encodes sphinganine C4-hydroxylase. The deletion of *FgSUR2* results in a defect in toxisome formation, leading to a significant reduction in DON biosynthesis [139]. FgCdc25 is characterized as the only Ras GTPase guanine nucleotide exchange factors (RasGEFs) protein in *F. graminearum*, and an *fgcdc25* mutation led to reduced toxisome formation and DON production [140]. *FgMYO1*, encoding a class I myosin, interacts with Tri1 and actin in *F. graminearum*. Toxisome formation is significantly reduced when FgMyo1 is inhibited by the small molecule phenamacril or when actin polymerization is disrupted by latrunculin A [141]. In *F. graminearum*, FgMyo1 and Tri1 directly interact with FgCapA and FgCapB, which are actin-capping proteins (CAPs). The mutants of Δ*FgcapA* and Δ*FgcapB* significantly disrupt toxisome formation and DON production [55]. In *F. graminearum*, the assembly of the functional toxisome relies on the α1-β2 tubulin heterodimer as the supporting structure [142]. 

### 3.4. DON Production and Plant Infection Are Affected by Environment Factors

Besides regulators that are specific to certain pathways, the biosynthesis of the DON toxin is also affected by various host and environmental factors (Figure 3). These factors, known as global regulators, include light, carbon, nitrogen, and pH [117]. Light controls the synthesis of trichothecenes through the regulation of the velvet complex. When the velvet complex is disrupted, it leads to a notable decrease in the production of DON [143,144]. The studies have revealed that sucrose is more effective at stimulating trichothecene production compared to glucose [145,146]. Polyamine biosynthesis is crucial for both plants and their pathogens, as it plays a significant role in enhancing stress tolerance and pathogenicity [147]. The infection of *F. graminearum* in wheat heads triggers the activation of pathways involved in the production of polyamines, which in turn triggers the biosynthesis of DON [148]. Deletion of *FgSPE3*, a gene involved in spermidine biosynthesis in *F. graminearum*, shows significantly decreased production of the DON and weak virulence in host plants [149]. FgAreA, a master regulator of nitrogen assimilation, modulates DON biosynthesis and undergoes nuclear translocation under nitrogen-limiting conditions or in response to putrescine [150]. Deletion of *fgareA* abrogates *TRI5*, *TRI6,* and *TRI10* expression and attenuates DON production upon arginine stimulation [151]. The acidic environment is essential for the transcription of *TRI* genes and the production of trichothecenes in *F. graminearum*, aligning with the acidification of the extracellular pH during fungal cultivation in mycotoxin-inducing media [117,152]. Conversely, neutralizing or alkalizing the environment inhibits trichothecene production and suppresses *TRI* genes [153]. In *F. graminearum*, FgPac1 serves as a negative regulator of trichothecene production. The mutant Δ*fgpac1* displays stunted growth in neutral and alkaline pH environments, but demonstrates accelerated *TRI* gene activation and trichothecene buildup in acidic conditions [154]. When *F. graminearum* infects a host, it causes the host to create an alkaline environment. This leads to FgPacC being cleaved into its functional form, called FgPacC30 [155]. 

Defense-related H_2_O_2_ generated in plants also contributes to the biosynthesis of DON during infection [156]. In the biotrophic stage of *F. graminearum* infection, the host plant is stimulated to produce a significant amount of H_2_O_2_ quickly. The additional H_2_O_2_ triggered by salicylic acid (SA) signaling can be advantageous for the fungus by promoting DON production [157]. When *F. graminearum* culture is exposed to either external H_2_O_2_ or the fungicide prothioconazole, which induces H2O2, the *TRI4* and *TRI5* genes are expressed at higher levels [158]. The stress-related transcription factor FgSkn7 is conscientious for H_2_O_2_-induced *TRI* gene expression. Mutants of *fgskn7* show decreased DON production and defection of *TRI* gene expression induced by H_2_O_2_ [70].

## 4. Perspectives

### 4.1. Disease Control Based on Virulence Gene

These genes summarized above are intimately involved with important stages of *F. graminearum*, and provide new additional sources for FHB control. Host-induced gene silencing (HIGS) and spray-induced gene silencing (SIGS) are emerging biotechnological approaches that use double-stranded RNA (dsRNA) to target essential fungal genes and suppress their expression. Several studies have demonstrated that HIGS and SIGS can effectively reduce FHB symptoms and mycotoxin accumulation by targeting genes involved in fungal growth, virulence, and toxin biosynthesis [159,160]. HIGS and SIGS offer several advantages over other control methods, such as specificity, durability, safety, and compatibility with existing breeding programs. Furthermore, the use of mycovirus-induced hypovirulence also shows promise in managing fungal diseases. Recently, a VIGS (virus-induced gene silencing) vector, p26-D4, derived from *F. graminearum* gemytripvirus 1 (FgGMTV1), has been effectively developed to transform the cereal FHB pathogen into a less virulent strain [161]. The p26-D4-VIGS system offers a novel approach for managing FHB and presents an extra method for preventing fungal diseases in various crops [162].

### 4.2. Molecular Design Breeding Based on F. graminearum Effectors

Fungal effectors, serving as vital tools for infection, target a wide array of plant genes, such as proteins involved in signal transduction, metabolic pathways, and plant immunity. These effectors play crucial roles in manipulating plant responses and facilitating fungal colonization by interfering with various aspects of plant physiology and immunity [108]. As more secreted proteins are characterized in *F. graminearum*, utilizing advanced tools such as the CRISPR/Cas9 system could enable the development of new, FHB-resistant wheat varieties. Some effectors interact with susceptibility genes to promote the expansion of *F. graminearum*, and disrupting these susceptibility genes through gene editing would probably increase the resistance of wheat to FHB. In contrast, some effectors decrease plant defense responses by targeting resistance genes. Overexpressing these resistance genes may also achieve the effect of FHB resistance [117].

## Data Availability

No new data were created or analyzed in this study. Data sharing is not applicable to this article.

## Supplementary Materials

The following supporting information can be downloaded at: https://www.mdpi.com/article/10.3390/genes15040475/s1, Table S1: Genes involved in sexual reproduction, Table S2: Genes involved in DON production.

## Abbreviations

FHB: Fusarium head blight; RH: runner hyphae; IC: infection cushions; IH: invasive hyphae; CAZymes: carbohydrate-active enzymes; DON: deoxynivalenol; QTL: quantitative trait loci; TFs: transcription factor genes; MAT: mating type; GPCRs: G protein-coupled receptors; RGS: regulator of G protein signaling; MAPK: mitogen-activated protein kinase; CWI: cell wall integrity; STRIPAK: striatin-interacting phosphatases and kinases; CAPs: capping proteins; MARKs: microtubule affinity-regulating protein kinases; Csn: COP9 signalosome; ROA: ORF round ascospore; RIP: repeat induced point mutation; MSUD: meiotic silencing by unpaired DNA; RNAi: RNA interference; dsRNA: double-stranded RNA; RISC: RNA-induced silencing complex; milRNAs: microrna-like RNAs; SSPs: small secreted proteins; GH12: glycoside hydrolase 12; RXEG1: response to XEG1; CWDEs: cell wall-degrading enzymes; CFEM: cysteine-rich common in fungal extracellular membrane; CEBiP: chitin elicitor-binding protein; TOR: target of rapamycin; H3K4me: histone 3 lysine 4 methylations; HAT: histone acetyltransferase; HDAC: histone deacetylase; FPP: farnesyl pyrophosphate; TDN: trichodiene; CAL: calonectrin; SAGA: Spt-ada-gcn5-acetyltransferase; lncRNA: long non-coding RNA; OSER: organized smooth endoplasmic reticulum; RasGEFs: Ras gtpase guanine nucleotide exchange factors; CAPs: capping proteins; SA: salicylic acid; HIGS: host-induced gene silencing; SIGS: spray-induced gene silencing; dsRNA: double-stranded RNA; VIGS: virus-induced gene silencing.
