# Supplementary material for: Advances in Understanding Fusarium graminearum: Genes Involved in the Regulation of Sexual Development, Pathogenesis, and Deoxynivalenol Biosynthesis
Source: Genes (Basel). 2024 Apr 9;15(4):475. doi: 10.3390/genes15040475 (PMC11050156; doi:10.3390/genes15040475)
Supplement: Supplementary file 1 [file genes-15-00475-s001.zip › Supplementary Table S1.pdf]

Table S1. Genes involved in sexual reproduction

	Phenotypes of deletion mutants	Genes	Proteins
Genes involved in sexual reproduction	No perithecium formation	*GIP1*	Non-pheromone G protein-coupled receptor
		GPA1	Heterotrimeric G proteins
		FgFLBA	Regulators of G protein signaling
		MGV1	MAPK
		GPMK1	
		HOG1	
		FgSTE12	Transcription factors
		FgMCM1	
		RAS2	GTPase
		PKR	cAMP-PKA
		FGK3	Glycogen synthase kinase
		FgVELB	Velvet protein complex
		FgEPS1	Protein disulfide isomerase
		FgAP1σ	AP1 complex
		FgDBR1	RNA lariat debranching enzyme
		Fgporin	Mitochondrial porin
		FgERV14	Endoplasmic reticulum (ER) cargo receptor
		FgBEM1	Phox homology domain-containing proteins
		SGH1	Serine/arginine (SR)-like protein
		FgExosc1	Components of the RNA exosome complex
	Reduced production of perithecia	*MES1*	Implicated in cell-surface organization
		FgSFL1	Transcription factors
		FgCAPA and *FgCAPB*	Actin capping proteins
		GzSNF1	Sucrose nonfermenting 1 protein kinase
	Delayed development of perithecia	*FgMET3* and *FgMET14*	Synthesis of cysteine and methionine
		FgATF1	Transcription factors
	Defective in ascospore formation	*GIA1*	Non-pheromone G protein-coupled receptor
		FgSWI6	Transcription factor
		FGP1	
		FgATF1	
		CPK1	Catalytic subunit of PKA
		FgExoscA	Components of the RNA exosome complex

		FgDBF2(Fg08635)	Protein kinase
		FgSWE1(Fg10228)	
		Fg08468, Fg07344, Fg06878 and *Fg10095*	
		FgKIN1	
		FgCSN12	Subunit of cop9 signalosome
		FgBUD14	Homology to yeast bud14
		GzSNF1	Sucrose nonfermenting 1 protein kinase
		ZIF1	B-zip transcription factor
		FgLEU1	Isopropylmalate isomerase
	Defective in ascospore release	*Fg01506, Fg13318, Fg08906, Fg01842, Fg06957, Fg10095*	Protein kinase
		CCH1	Calcium ion channel gene
		FgSRP1	SR (serine/arginine-rich) protein
		FgATF1	Stress-related transcription factor
		FgSP1	Transporter
		GEA1	
		ROA	
