# Supplementary material for: Advances in Understanding Fusarium graminearum: Genes Involved in the Regulation of Sexual Development, Pathogenesis, and Deoxynivalenol Biosynthesis
Source: Genes (Basel). 2024 Apr 9;15(4):475. doi: 10.3390/genes15040475 (PMC11050156; doi:10.3390/genes15040475)
Supplement: Supplementary file 1 [file genes-15-00475-s001.zip › Supplementary Table S2.pdf]

Table S2. Genes involved in DON production

	Phenotypes of deletion mutants	Genes	Proteins
Genes involved in DON production	Decreased DON production	*CPK1*	Catalytic subunits of cyclic AMP (camp)-dependent (PKA)
		FgCAP1	Adenylate-binding protein
		FgBCK1	Protein kinase
		FgMKK1	
		FgMGV1	MAPK
		FgSTE11-STE7-GPMK1	MAPK
		FgHOG1	MAPK
		FgSHO1	Transmembrane protein
		FgSTE50	Transcription factor
		FgOS1	Response regulators
		FgRRG1	Response regulators
		FgATF1	Response factor
		FgSTUA	Transcription factor
		FgPEX13, *FgPEX14*	Docking machinery components
		FgPEX33	Filamentous fungal specific peroxin
		HEP1	Heterochromatin protein
		KMT6	Histone acetyltransferases
		FgGCN5	
		ELP3	
		FgSAS3	
		FNG1 and *FNG3*	Inhibitor of Growth (ING) proteins
		FgAREA	Master regulator of nitrogen assimilation
		FgSKN7	Transcription factor
		FgSPE3	Involved in spermidine biosynthesis
		FgPRB1	Subtilisin-like protease
	Increased DON production	*PDE2*	cAMP phosphodiesterase
		FgPAC1	Transcription factor
		PKR	Regulatory subunit of PKA
	Defective in formation of toxisomes	*FgSUR2*	Sphinganine C4-hydroxylase
		FgCDC25	Ras GTPase
		FgMYO1	Class I myosin
		FgCAPA and *FgCAPB*	Actin capping proteins
